# Epidemiology of Hepatitis B and C Virus Infections and Liver Cancer in Vietnam

**DOI:** 10.5005/jp-journals-10018-1130

**Published:** 2015-01-06

**Authors:** Son Huy Do

**Affiliations:** Department of Health, Binh Thuan Medical College, Binh Thuan Province, Vietnam

**Keywords:** Epidemiology, Hepatitis B virus, Hepatitis C virus, Liver cancer, Vietnam.

## Abstract

Vietnam has been a highly endemic country of hepatitis B virus (HBV) and hepatitis C virus (HCV) infections with high hepatitis B surface antigen (HBsAg) rate (8.8-19.0%) and high anti-HCV rate (1.0-3.3%) among general population. High mortality rates of liver cancer were also estimated from 14.8 to 23.7 per 100,000. Recently, the coverage of universal HB vaccination for infants has not been high as expected while there have been no other national programs against HBV and HCV infections yet. The burden of HBV and HCV-related diseases is predicted to remain a significant health problem in next decade.

**How to cite this article:**

Do SH. Epidemiology of Hepatitis B and C Virus Infections and Liver Cancer in Vietnam. Euroasian J Hepato-Gastroenterol 2015;5(1):49-51.

## INTRODUCTION

In 2012, International Agency for Research on Cancer, World Health Organization (WHO)^1^ estimated that Vietnam was one of the countries with the highest mortality from liver cancer, which is mostly attributed to persistent infections with hepatitis B virus (HBV) and hepatitis C virus (HCV).^2^ As there has been neither nationwide study nor national registration system in viral hepatitis and liver cancer, recent published researches in different regions of Vietnam play an important role in partly clarifying epidemiological characteristics of HBV and HCV infections as well as liver cancer in this country.

## HEPATITIS B VIRUS INFECTION

In accordance with an estimation of WHO,^3^ prevalence of hepatitis B surface antigen (HBsAg) in Vietnam was as high as 8% or over, some population-based studies in different provinces in northern Vietnam found HBsAg rates of 18.8,^4^ 19.0^5^ and 8.8%.^6^ Recently, a sero-epidemiological research^7^ with randomly sampled participants among general population in Binh Thuan Province, southern Vietnam revealed a high HBsAg rate of 15.3% and particularly high rate of hepatitis B core antibody (HBcAb) of 71.7%. Taken together, these evidences indicate that Vietnam is a highly endemic country of HBV infection. Furthermore, persistent HBV infection was predicted to remain endemic in next decade, despite the achievement of the universal HB vaccination program for infants.^8^ While no difference in HBV infection rate between genders was found, HBV exposure (i.e. the positivity for HBsAg and/or HBc Ab) was observed significantly higher with increased age groups.^5-7^ Although various HBsAg rates were shown in different regions of Vietnam, no obvious trend of regional distribution of HBV infection in this country was reported.

As for HBV genotypic distribution in Vietnam, recent study among general population found that the most common HBV genotype was B (75.3%), followed by C (11.7%).^7^ Comparable distribution of genotypes B and C were also observed.^9,10^ However, other hospital-based researches showed the predominance of genotype C^11,12^ among patients with HBV infection. It was reported that genotype B was common among asymptomatic HBV-infected cases while genotype C was predominant in patients with liver cirrhosis and hepatocellular carcinoma (HCC).^13^ Thus, different study population might be one of the reasons why different HBV genotypic distributions were found in Vietnam.

It was well-known that the main route of HBV infection in endemic countries was mother-to-child transmission, followed by horizontal transmission during early childhood.^14^ Interestingly, horizontal HBV transmission was supposed to be more frequent than previously thought in Vietnam.^7^

## HEPATITIS C VIRUS INFECTION

Epidemiological data on HCV infection in Vietnam might be even more limited than that of HBV infection. Prevalence of HCV infection in Vietnam was recently estimated as 2%^15^ while an earlier study found an anti-HCV rate of 1°% in northern Vietnam.^16^ However, in 2012, a higher rate of anti-HCV of 3.3% was revealed among general population in southern Vietnam.^7^ The most updated data were in concordance with the current report of Center for Disease Control and Prevention of United State that prevalence of HCV infection Vietnam is as high as 2.0 to 2.9%.^17^ No significant difference in HCV infection rate between genders was reported^7,16^ whereas age groups of 50 or over were found to have high-risk of anti-HCV seropositivity.^7^

Some studies on HCV genotypic distribution in Vietnam also showed different results. A study of 2000 HCV RNA positive samples from many hospitals in Ho Chi Minh city^18^ reported that genotype 1b was the most common (58%), followed by genotype 6a (17%). By contrast, other studies showed the predominance of genotype 6a among blood donors (37.1%)^19^ and among general population (55.6%).^7^ This discrepancy might be explained by the fact that HCV genotype 1b is more related to progressive liver diseases in comparison with other HCV genotypes.^20^

## LIVER CANCER

Liver cancer has been the most frequent cause of cancer death in Vietnam.^1^ High mortality rates of liver cancer were reported from 14.8 to 23.7 per 100,000.^1,21,22^

In South-east Asian Region, where Vietnam belongs to, it was estimated that 65 and 18% of HCC cases were caused by persistent infection with HBV and HCV respectively 2. A study in central Vietnam showed that approximately 90% of patients with HCC had evidence of HBV, and one-seventh of them were related to HCV.^15^ Although published studies on relationship between HCC and HBV and/or HCV infection in Vietnam are rare, HBV and HCV have been considered the most important etiology of HCC in Vietnam.

So far, there has been regrettably no nationwide comprehensive approach to the HBV and HCV-related liver diseases, including HCC, in Vietnam^15^ except the universal HB vaccination program for infants started since 2002. Unfortunately, the coverage of HB vaccination has fluctuated and dropped to under 60% in 2013.^23^ Hence, to reduce the burden of HBV and HCV-related liver diseases, it is crucial to raise the coverage of the universal HB vaccination for infants as well as to promote other preventive measures against horizontal transmission of both HBV and HCV.
